# CREG1 heterozygous mice are susceptible to high fat diet-induced obesity and insulin resistance

**DOI:** 10.1371/journal.pone.0176873

**Published:** 2017-05-01

**Authors:** Xiaoxiang Tian, Chenghui Yan, Meili Liu, Quanyu Zhang, Dan Liu, Yanxia Liu, Shaohua Li, Yaling Han

**Affiliations:** 1 Cardiovascular Research Institute and Department of Cardiology, General Hospital of Shenyang Military Region, Shenyang, China; 2 Cardiovascular Center for Translational Medicine of Liaoning Province, Shenyang, China; 3 Cardiovascular Core Lab for Translational Medicine of Liaoning Province, Shenyang, China; 4 Department of Surgery, Robert Wood Johnson Medical School, Rutgers-the State University of New Jersey, New Brunswick, United States of America; Thomas Jefferson University, UNITED STATES

## Abstract

Cellular repressor of E1A-stimulated genes 1 (CREG1) is a small glycoprotein whose physiological function is unknown. In cell culture studies, CREG1 promotes cellular differentiation and maturation. To elucidate its physiological functions, we deleted the *Creg1* gene in mice and found that loss of CREG1 leads to early embryonic death, suggesting that it is essential for early development. In the analysis of *Creg1* heterozygous mice, we unexpectedly observed that they developed obesity as they get older. In this study, we further studied this phenotype by feeding wild type (WT) and Creg1 heterozygote (*Creg1*^*+/-*^) mice a high fat diet (HFD) for 16 weeks. Our data showed that *Creg1*^*+/-*^ mice exhibited a more prominent obesity phenotype with no change in food intake compared with WT controls when challenged with HFD. *Creg1* haploinsufficiency also exacerbated HFD-induced liver steatosis, dyslipidemia and insulin resistance. In addition, HFD markedly increased pro-inflammatory cytokines in plasma and epididymal adipose tissue in *Creg1*^*+/-*^ mice as compared with WT controls. The activation level of NF-κB, a major regulator of inflammatory response, in epididymal adipose tissue was also elevated in parallel with the cytokines in *Creg1*^*+/-*^ mice. These pro-inflammatory responses elicited by CREG1 reduction were confirmed in 3T3-L1-derived adipocytes with CREG1 depletion by siRNA transfection. Given that adipose tissue inflammation has been shown to play a key role in obesity-induced insulin resistance and metabolic syndrome, our results suggest that *Creg1* haploinsufficiency confers increased susceptibility of adipose tissue to inflammation, leading to aggravated obesity and insulin resistance when challenged with HFD. This study uncovered a novel function of CREG1 in metabolic disorders.

## Introduction

Obesity is a growing epidemic with tremendously increased prevalence over the last 30 years worldwide [1]. Obesity is a major independent risk factor for metabolic diseases including insulin resistance, type 2 diabetes (T2D), coronary artery disease, stroke, nonalcoholic fatty liver disease and certain cancers [2–5]. Previous studies have demonstrated that a state of chronic, low-grade inflammation characterized by abnormal cytokine production and activation of inflammatory signaling pathways in adipose tissue is an initial event during pathogenesis in obesity [6–8]. Although it is well established that adipose inflammation deteriorates cellular metabolism and impairs insulin signaling during obesity [9], the underlying mechanism are largely unknown.

Cellular repressor of E1A-stimulated genes (CREG1) was firstly reported in 1998 by Gill et al as a transcription repressor that antagonizes E1A-induced transcription activation [10]. Subsequent studies showed that it is a small glycoprotein with 220 amino acid residues to be either secreted outside the cell or reside in intracellular membrane compartments [11, 12]. Human CREG1 has three glycosylation sites which are required for its binding to its putative receptor mannose 6 phosphate/insulin like growth factor 2 receptor (IGF2R) [13, 14]. Our previous studies showed that CREG1 plays important roles in maintaining a mature, differentiated phenotype of vascular smooth muscles cells, endothelial cells and cardiomyocytes in vitro [15–18]. In addition, over-expression of CREG1 protected mice from atherosclerosis, pressure over-loaded ventricular remodeling and myocardial fibrosis in vivo [19–21]. Of note, we also found that over-expression of CREG1 inhibited tumor necrosis factor α (TNF-α)-induced apoptosis in mesenchymal stem cells, protected endothelial cell from TNF-α-induced hyper-permeability, and attenuated macrophage inflammation [19, 22, 23]. The nuclear factor-κB (NF-κB) pathway and autophagy seem to mediate these anti-inflammatory effects [19, 23]. Based on these findings, we tested the hypothesis that CREG1 might suppress development of obesity and insulin resistance by inhibiting inflammatory responses. In this study, we show that haplodeficiency of *Creg1* exacerbated high fat diet (HFD)-induced obesity, insulin resistance and dyslipidemia without affecting food consumption, suggesting an imbalance between calorie intake and expenditure. In addition, HFD triggered systemic and adipose tissue inflammation in *Creg1*^***+/-***^ mice, possibly through activation of the NF-κB pathway. To the best of our knowledge, this is the first demonstration that CREG1 negatively regulates the pathogenesis of obesity and related metabolic dysfunctions. These results not only provide new insights into the function of CREG1 but also help us to better understand the molecular mechanisms of obesity.

## Materials and methods

### Ethics statement

All animal experiments were performed in accordance with the Principles of Laboratory Animal Care formulated by the National Society for Medical Research and the Guide for the Care and Use of Laboratory Animals (NIH Publication 86–23, 1985 revision). Animal care and procedures were approved by the Ethics Committee on the Care and Use of Laboratory Animals of General Hospital of Shenyang Military Region.

### Animal and diets

*Creg1* heterozygote (*Creg1*^*+/-*^) mice in the C57BL/6J background were obtained from the National Resource Center of Model Mice (Nanjing University, Nanjing, China) as previously described [24]. *Creg1*^*+/-*^ mice were verified by genotyping and had been backcrossed at least 10 times into the C57BL/6J background before use. Wild type (WT) littermates were used as control. All mice were housed in specific pathogen-free animal facility at ambient temperature of 23 ± 2°C with a dark-light cycle of 12–12 hrs and ad libitum access to food and water. Mice of 8 weeks of age were fed with either control normal diet (ND; 10% of total calories from fat, 70% from carbohydrate and 20% from protein, D12450J, Research Diets Inc., New Brunswick, NJ) or high fat diet (HFD; 60% of total calories from fat, 20% from carbohydrate and 20% from protein, D12492, Research Diets Inc.) for 16 weeks.

Food consumption and body weights were measured daily and weekly respectively. After 16 weeks of feeding, the mice were fasted overnight prior to anesthesia (2.5% isoflurane). Mice were euthanized with 5% isoflurane followed by exsanguination of left common carotid artery to harvest blood samples. Plasma samples were obtained by centrifugation at 3000 rpm for 15 min and stored at -80°C for further biochemical analysis. The fat pads (inguinal, epididymal and peri-renal) and liver were dissected, removed and weighted. A portion of the epididymal adipose tissue and liver was fixed in 4% paraformaldehyde for histological analysis and the other portion was immediately snap-frozen in liquid nitrogen and stored at -80°C for RNA and protein preparation. All animal experiments were approved by Ethics Committee on the Care and Use of Laboratory Animals of General Hospital of Shenyang Military Region.

### PCR genotyping

Genomic DNA from tail tips of WT and *Creg1*^*+/-*^ mice was extracted by a One Step Mouse Genotyping Kit (Vazyme Biotech, Nanjing, China) and used as template for amplification of WT and Mutant Creg1 gene by PCR. Since the exons 2 and 3 of one *Creg1* allele were replaced with a neomycin-resistant (Neo^R^) cassette in *Creg1*^*+/-*^ mice, *Creg1* heterozygosity was confirmed by detection of both WT and Neo^R^ cassette. Two primer sequences used for genotyping were 5’-TGTCGGGAACTGTGACCAAG-3’ (forward) and 5’-CTTTAGGTCCACCAAAGTAG-3’ (reverse) for the WT Creg1, and 5’-CTCAGCCTTGGGGGTGCTGGGAAGA-3’ (forward) and 5’-TCGTCGTGACCCATGGCGATGCCTG-3’ (reverse) for mutant *Creg1*, respectively.

### Induction of adipocyte differentiation and knockdown of *Creg1*

For induction of adipocyte differentiation, 3T3-L1 cells purchased from ATCC (Manassas, VA) were induced in differentiation DMEM medium containing 10% fetal calf serum (ThermoFisher, Grand Island, NY), 1% penicillin-streptomycin, 0.5 mmol/L iso-butyl-methyl xanthine, 1 μmol/L dexamethasone, 10 μmol/L pioglitazone, and 1.7 μmol/L insulin (Sigma, St. Louis, MO) for 2 days. Cells were then incubated in maintenance DMEM medium containing 10% fetal calf serum, 1% penicillin-streptomycin and 1.7 μmol/L insulin for 6 days for lipid accumulation.

For Creg1 knockdown, the differentiated adipocytes were transfected with 25 nmol/L Creg1 siRNA (Santa Cruz, Dallas, Texas) by Fugene6 (Roche, Shanghai, China) for 48 hrs, a scrambled siRNA (Santa Cruz) was used as control. Creg1 knockdown efficiency was assessed by Western blotting. When liposaccharide (LPS) treatment was needed, cells were exposed to 10 ng/ml LPS for 2 days after siRNA transfection. Medium was changed every other day.

### Histology of adipose tissue and liver

Epididymal adipose tissue and the liver dissected from mice were fixed in 4% paraformaldehyde for 48 hrs and embedded in paraffin after dehydration. Sections of 5 μm were cut with a microtome (Leica RM2235). Sections were deparaffinized and routinely stained with hematoxylin and eosin (H&E). Images were captured by a Leica microscope. Adipocyte size was measured in digital micrographs of about 300 cells per mouse (n = 8–10) from 8 randomly selected fields using ImageJ software (National Institutes of Health, Bethesda, MD, http://imagej.nih.gov/ij/, 1997–2012).

### Fasting blood glucose and insulin

After overnight fasting, blood samples were obtained by tail bleeding and blood glucose was measured weekly with a glucometer and glucose test strips (Roche). At the end of the experiment, plasma was obtained from the left common carotid artery and fasting blood glucose was measured with an automatic biochemical analyzer (Cobas 400 plus, Roche, Switzerland). Fasting insulin levels were determined using a mouse insulin ELISA kit (Thermo) according to the manufacturer’s instruction. Absorbance was measured at 450 nm and insulin levels were calculated by plotting a four-parameter logistic curve fit after generation of a standard curve. Insulin resistance was evaluated at the end of experiment by a homeostatic model assessment for insulin resistance (HOMA-IR): HOMA-IR = fasting glucose level (mmol/L) × fasting insulin level (mIU/L) / 22.5.

### Glucose tolerance test (GTT) and insulin tolerance test (ITT)

For the GTT or ITT, mice fasted overnight were injected intraperitoneally with glucose (2 g/kg body weight) or insulin (0.75 U/kg body weight) (Novonordisk, Copenhagen, Denmark), respectively. Blood glucose was measured at indicated times (0, 15, 30, 60, and 120 min after glucose or insulin administration). Glucose tolerance was calculated by area under the curve (AUC) and insulin sensitivity was calculated by inverse AUC.

### Blood biochemical analysis

Plasma samples were analyzed for total cholesterol (TG), triglyceride (TC), low density lipoprotein-cholesterol (LDL-C), free fatty acid (FFA), tumor necrosis factor α (TNF-α), interleukin 6 (IL-6), monocyte chemoattractant protein 1 (MCP-1), adiponectin and leptin by using Cholesterol Quantitation Kit (Sigma), Triglyceride Quantification Kit (Abcam), HDL and LDL/VLDL Cholesterol Assay Kit (Abcam), Free Fatty Acid Quantification Kit (Abcam), Mouse Tumor Necrosis Factor α ELISA Kit (Sigma), Mouse IL-6 ELISA Kit (Sigma), Mouse MCP-1 / CCL2 ELISA Kit (Sigma), Mouse Adiponectin ELISA Kit (Abcam) and Mouse Leptin ELISA Kit (Abcam) according to the manufacturers’ instructions.

### Western blotting

Tissues were homogenized in RIPA lysis buffer supplemented with protease and phosphatase inhibitors (Thermo). Cells were lysed in either RIPA buffer or extraction buffer provided by a Nuclear Extraction Kit (Abcam) to obtain total proteins or nuclear proteins. Western blotting was performed as described previously [25]. Primary antibodies against Creg1 (Abcam), phosphorylated (p)- NF-κB (p65), NF-κB, p-IκBα and IκBα (Cell Signaling Technology, Danvers, MA) were used with 1:1000 dilution. Antibodies against β-actin (Abcam) and Lamin B (Sigma) were used as a loading control for total proteins or nuclear proteins, respectively. After blotting, films were scanned and the bands of interest were quantified by densitometry using Image J software (National Institutes of Health).

### Real-time PCR analysis

Total RNA from adipose tissue or differentiated 3T3-L1 cells were extracted using the TRIzol solution (Thermo). One μg of total RNA was reverse-transcribed using the PrimeScript RT reagent kit (Takara, Dalian, China) to obtain the complimentary DNA. SYBR Green PCR Master Mix (Applied Biosystems, Foster City, CA) was prepared and real-time PCR was performed using ABI 7300 Real-Time PCR Systems as previously described [20]. Each sample was amplified in triplicate. The signal of the PCR products was normalized to GAPDH transcripts. The primer pairs are listed in S1 Table.

### Lipolysis analysis

Glycerol and free fatty acids released into the culture medium of differentiated 3T3 L1 cells were measured to evaluate lipolysis. In brief, differentiated 3T3 L1 cells with or without CREG1 knockdown were treated with BMS-345541 (5 μmol/L, Sigma), an NF-κB pathway inhibitor, or vehicle (dimethyl sulfoxide, DMSO, Sigma) for 48 hours prior to lipolysis assessment. The glycerol level was determined by Lipolysis Assay Kit (Abcam) and the free fatty acid level was measured by Free Fatty Acid Quantification Kit (Abcam) according to the manufacturer’s instruction.

### Statistical analysis

All data are presented as means ± standard error of mean (SEM) and analyzed using SPSS software version 20.0 (IBM Corp., Armonk, NY). An unpaired Student t-test analysis was used for all data comparisons between *Creg1*^*+/-*^ and WT control mice fed ND or HFD. Significance in all tests was set at *P* < 0.05.

## Results

### *Creg1* haplodeficiency exacerbates HFD-induced obesity

**Fig 1:**
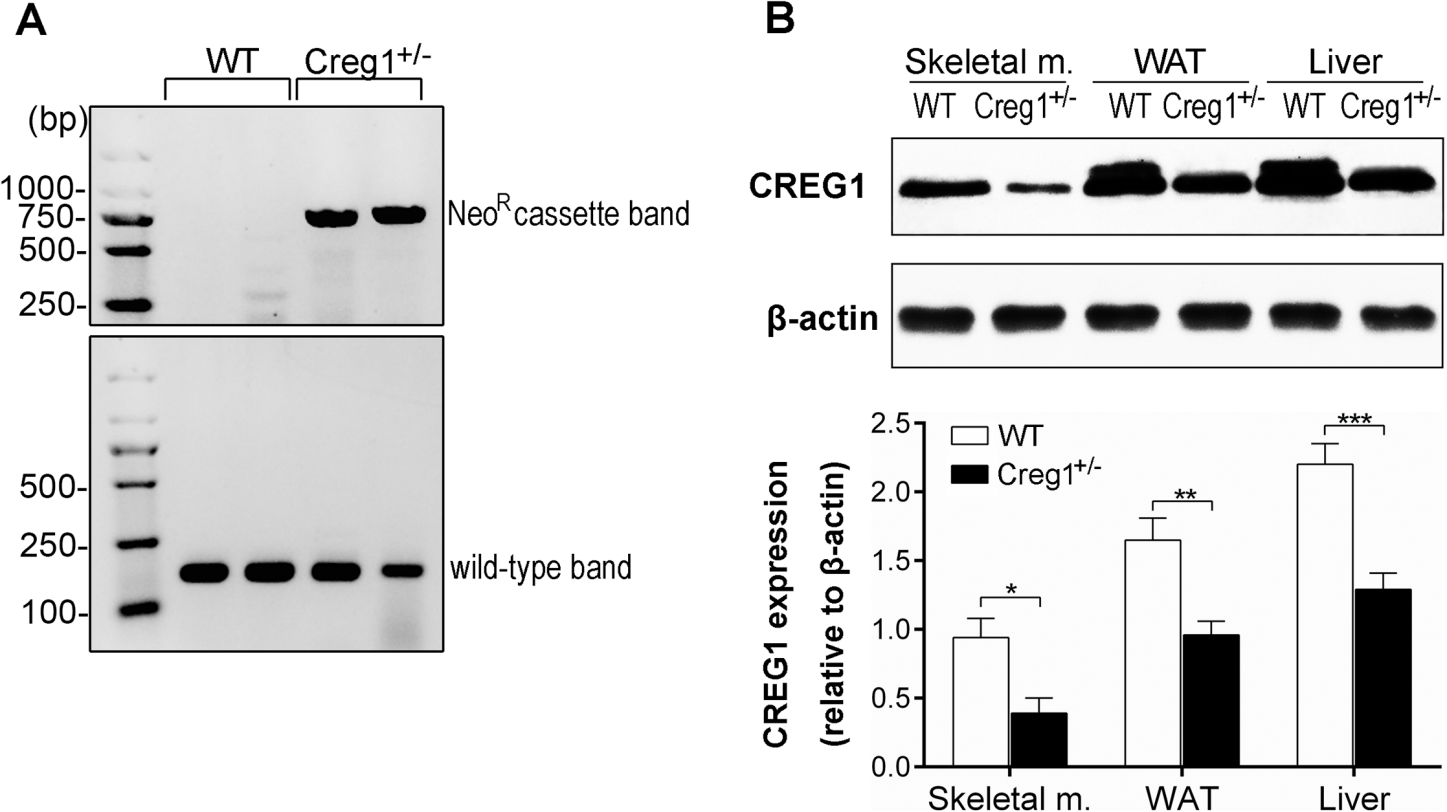
Verification of *Creg1*^*+/-*^ mice. (**A**): *Creg1*^*+/-*^ mice and their wild type (WT) littermates were genotyped by RT-PCR. The CREG1 gene in *Creg1*^*+/-*^ mice was disrupted by replacing exons 2 and 3 with a neomycin-resistant (Neo^R^) cassette. Representative genotyping image showed the wild type band of 168 bp amplicon and knockout (Neo^R^ cassette) band of 750 bp amplicon. (**B**): Skeletal muscle (m.), epididymal white adipose tissue (WAT) and Liver from WT and *Creg1*^*+/-*^ mice were assessed for *Creg1* expression by Western blotting and β-actin was used as a loading control. The lower panel showed the quantification of CREG1 intensity relative to β-actin (n = 3 from three independent experiments). Values are presented as means ± SEM. *P<0.05, **P<0.01 and ***P<0.001.

**Fig 2:**
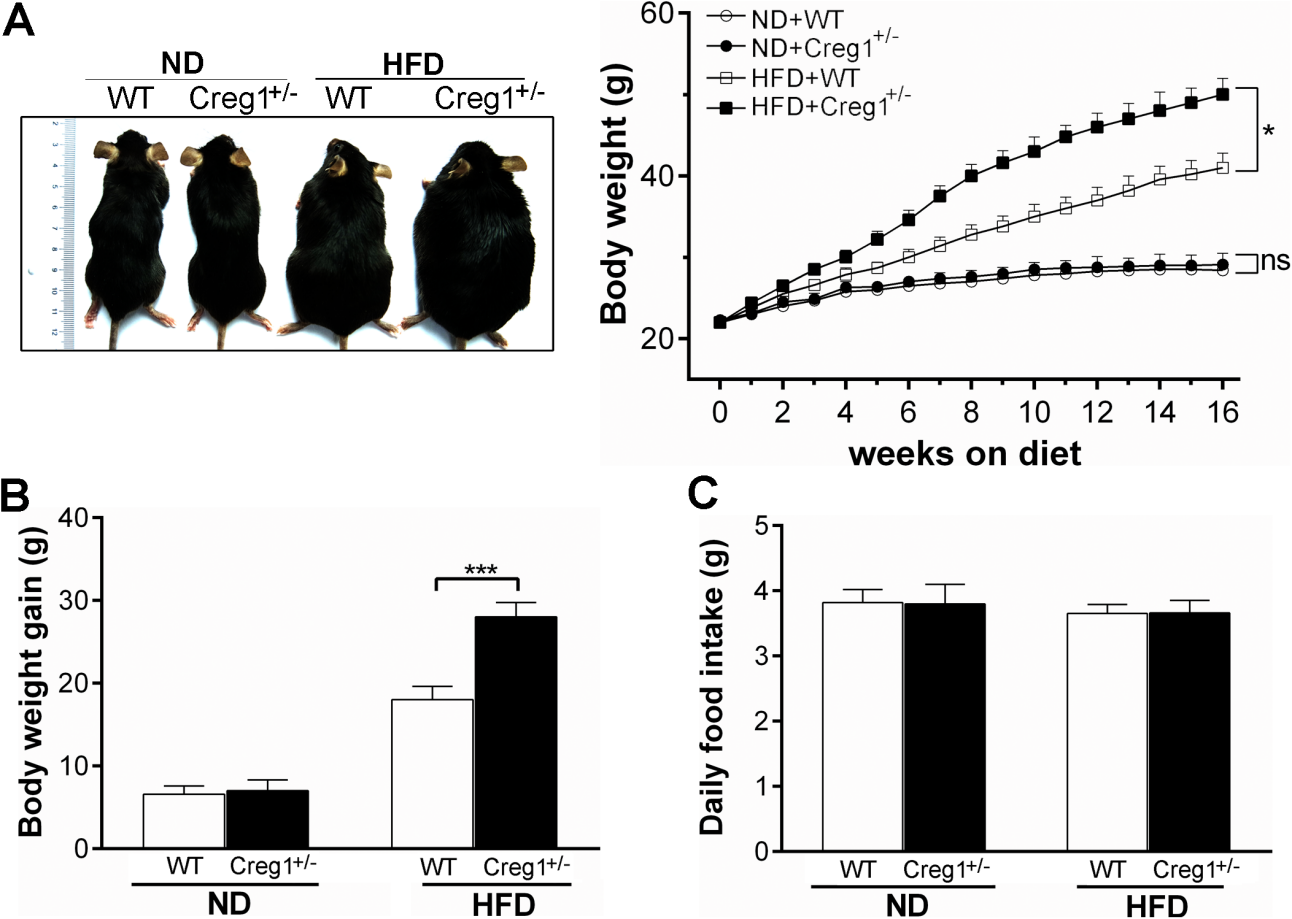
*Creg1*^+/-^ mice display increased weight gain with unchanged food intake on high fat diet (HFD). (**A**): Body weights of *Creg1*^*+/-*^ mice and their wild type (WT) littermates on 16 weeks of normal diet (ND) or HFD (n = 10). The left panel shows representative gross image. (**B**) Body weight gain of WT and *Creg1*^*+/-*^ mice after 16 weeks of ND or HFD (n = 10). (**C**) Average daily food intake of WT and *Creg1*^*+/-*^ mice during experiment (n = 10). Values are presented as means ± SEM. ns, no significance. *P<0.05 and ***P<0.001.

Our genetic analysis revealed that global knockout of *Creg1* gene leads to early embryonic death around E7.5 (unpublished data), so *Creg1*^*+/-*^ mice were used in this study. The *Creg1*^+/-^ mice were established as previously reported [24] and confirmed by PCR genotyping, which showed the expected 168 bp wild-type and the 750 bp knockout (Neo^R^ cassette) band (Fig 1A). Western blot analysis revealed that CREG1 expression was higher in white adipose tissue (WAT) than skeletal muscle but lower than the liver in WT mice (Fig 1B). Ablation of one allele of *Creg1* caused ~50% reduction of CREG1 expression in the above tissues (Fig 1B). To test whether the reduction of CREG1 affects fat metabolism, WT and *Creg1*^+/-^ mice were fed with either ND or HFD for 16 weeks. As expected, WT mice fed with HFD looked obese and gained more body weight than the ND controls (Fig 2A). Of note, *Creg1*^+/-^ mice showed a much more prominent obese phenotype and gained ~30% more body weight than WT mice on HFD. We observed no difference in body weight gain between WT and *Creg1*^+/-^ mice on ND (Fig 2A and 2B). To investigate whether the above findings were due to increased calorie consumption, we compared food intake in all mice. There was no difference in the average weight of daily food intake between WT and *Creg1*^+/-^ mice no matter which diet was fed (Fig 2C), demonstrating that calorie intake was similar in WT and *Creg1*^+/-^ mice fed with either ND (3.85 Kcal/g chow) or HFD (5.24 Kcal/g chow).

**Fig 3:**
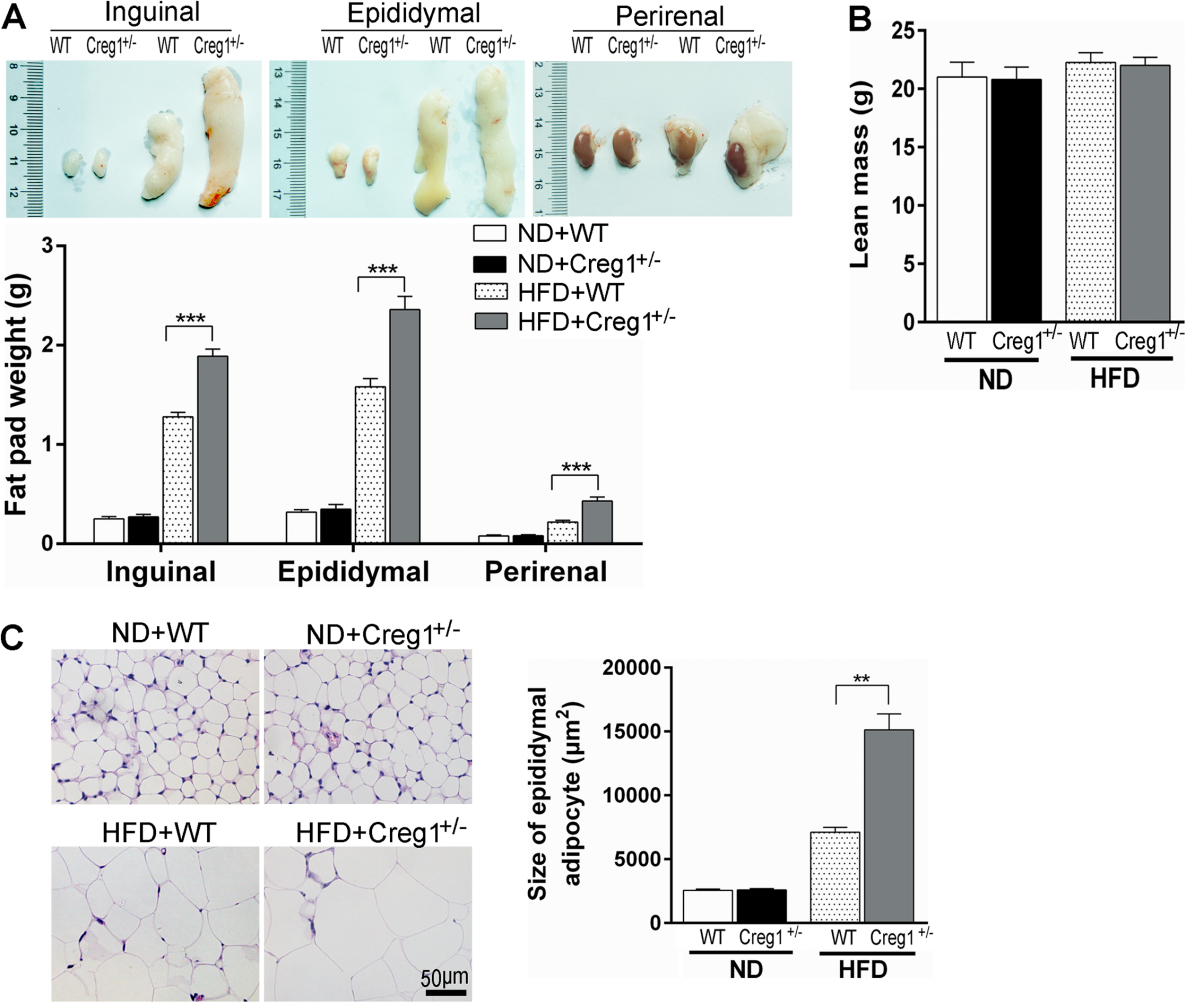
*Creg1*^*+/-*^ mice show increased susceptibility to high fat diet (HFD)-induced adiposity. On 16 weeks of normal diet (ND) or HFD, wild type (WT) or *Creg1*^*+/-*^ mice were sacrificed for the following measurements. (**A**): Representative gross image (upper panel) and weight (lower panel) of inguinal, epididymal and peri-renal adipose tissue (n = 8 to 10). (**B**): Lean mass of WT and *Creg1*^*+/-*^ mice. (**C**): Representative histological image of H&E staining (left panel) and average area of adipocytes (right panel) from epididymal adipose tissues (n = 8 to 10). Values are presented as means ± SEM. **P<0.01 and ***P<0.001.

To determine the source of increased body weight gain in *Creg1*^+/-^ mice, we focused our analysis on WAT because fat accumulation was the most striking change in both genotypes after HFD feeding. The *Creg1*^+/-^ mice displayed more deposited fat in inguinal, epididymal and peri-renal WAT when fed with HFD compared to WT controls (Fig 3A). However, no difference was found in these mice on ND (Fig 3A). There was no difference in lean mass between WT and *Creg1*^+/-^ mice fed with either ND or HFD (Fig 3B). In addition, histology of epididymal WAT showed that the size of adipocyte was larger in *Creg1*^+/-^ mice than that of WT controls on HFD (~2 folds, *P*<0.01) (Fig 3C). These results suggested that the extra weight gain in *Creg1*^+/-^ mice induced by HFD was mainly derived from adipose tissues, which is one of the key characteristics of obesity. By contrast, no significant difference in fat deposition was found between *Creg1*^+/-^ and WT mice under ND, implying that reduction of CREG1 by half can still maintain a normal body weight if not challenged with HFD.

### *Creg1*^*+/-*^ mice on HFD develop aggravated dyslipidemia, liver steatosis, glycaemia and insulin resistance

**Fig 4:**
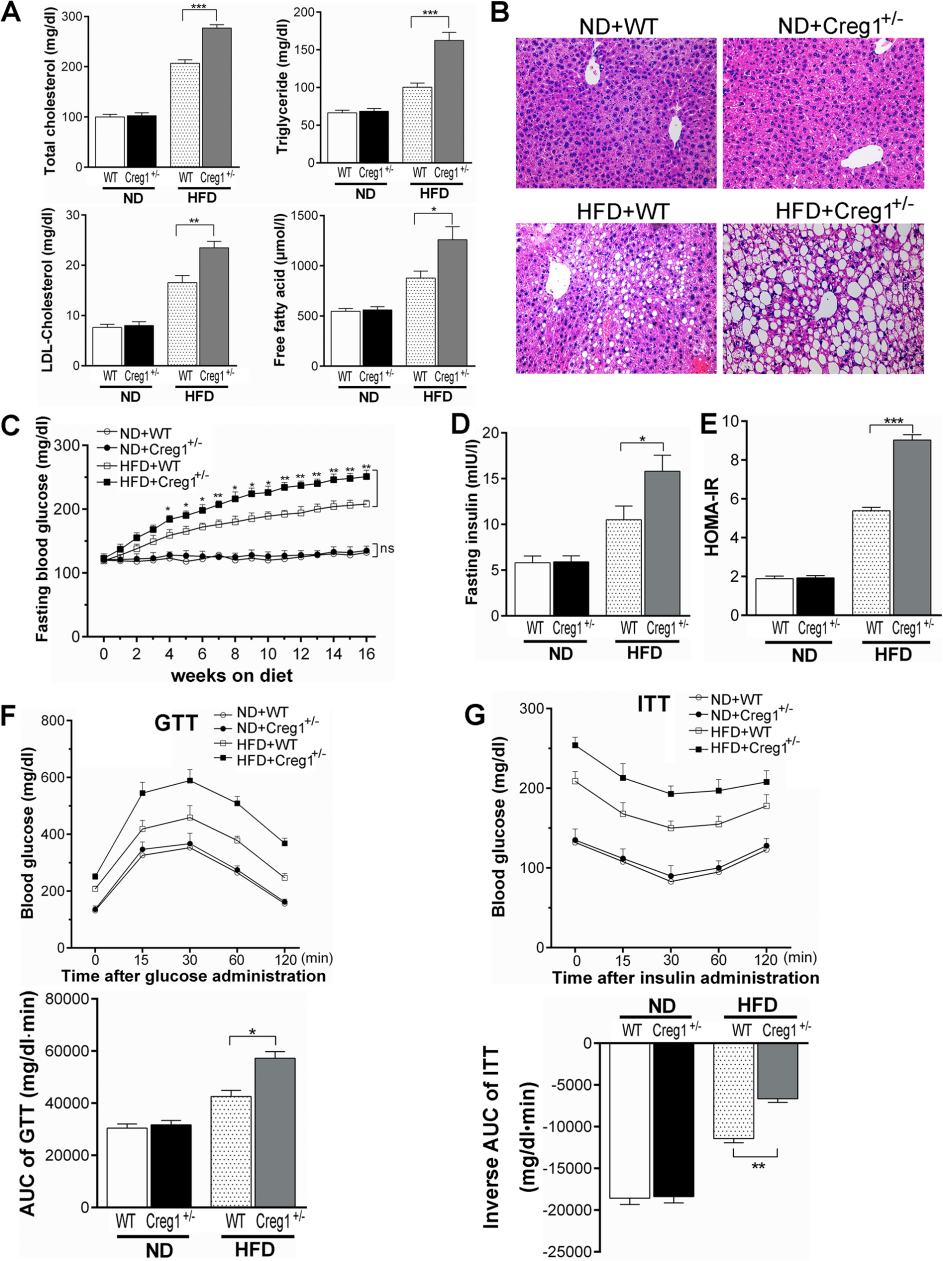
*Creg1*^*+/-*^ mice develop aggravated dyslipidemia, liver steatosis, glycaemia and insulin resistance on high fat diet (HFD). After 16 weeks of normal diet (ND) or HFD, (**A**): plasma lipid profile including total cholesterol (TC), triglyceride (TG), low density lipoprotein (LDL)-cholesterol and free fatty acid (FFA) of wild type (WT) and *Creg1*^*+/-*^ mice were measured by ELISA method (n = 8) and (**B**): liver steatosis was evaluated by H&E staining of paraffin-embedded sections (n = 8 to 10). (**C**): Fasting blood glucose, (**D**): fasting insulin concentration, and (**E**): homeostasis model assessment-insulin resistance [HOMA-IR = fasting glucose (mmol/l) × fasting insulin (mIU/l)/22.5] in WT and *Creg1*^*+/-*^ mice fed on ND or HFD for 16 weeks (n = 8). (**F**): Results of glucose tolerance test (GTT, upper panel) and quantification of area under curve (AUC) of GTT (lower panel) (n = 8). (**G**): Results of insulin tolerance test (ITT, upper panel) and quantification of inverse AUC of ITT (lower panel) (n = 8). Values are presented as means ± SEM. ns, no significance. *P<0.05, **P<0.01 and ***P<0.001.

Diet induced obesity is typically followed by systemic metabolic disorders including dyslipidemia, liver steatosis, glycaemia and insulin resistance, which further contributes to type 2 diabetes and cardiovascular diseases [26]. We firstly detected lipid profile in plasma. The level of TG, TC, LDL-C and FFA was elevated more in *Creg1*^+/-^ mice than that in WT controls on HFD (Fig 4A). In consistence with the changes of plasma lipids, the liver of *Creg1*^+/-^ mice on HFD displayed severe steatosis as indicated by more and larger lipid droplets in hepatocytes compared with WT controls (Fig 4B).

To examine whether insulin sensitivity is altered, fasting blood glucose was monitored weekly and insulin levels were detected at the end of the experiment. Under ND, blood glucose and insulin levels of *Creg1*^+/-^ mice were similar to those of WT controls (Fig 4C and 4D). In contrast, when fed with HFD, the glucose level in *Creg1*^+/-^ mice was significantly higher, beginning as early as 4 weeks of HFD feeding and continuing to the end of the experiment, and so was the plasma insulin level as compared with WT controls (Fig 4C and 4D). Furthermore, the HOMA-IR scores increased by nearly 1-fold in *Creg1*^+/-^ mice on HFD compared with their WT counterparts (Fig 4E). GTT and ITT were also performed at the end of the experiment to evaluate whole-body insulin sensitivity. Integrated plasma glucose concentration, as calculated by AUC, was more profoundly increased in *Creg1*^+/-^ mice as compared with WT controls on HFD but not ND (Fig 4F and 4G). These results indicate that insulin sensitivity is significantly impaired in *Creg1*^+/-^ mice fed with HFD.

### *Creg1*^*+/-*^ mice are sensitive to HFD-induced systemic and adipose tissue inflammation

**Fig 5:**
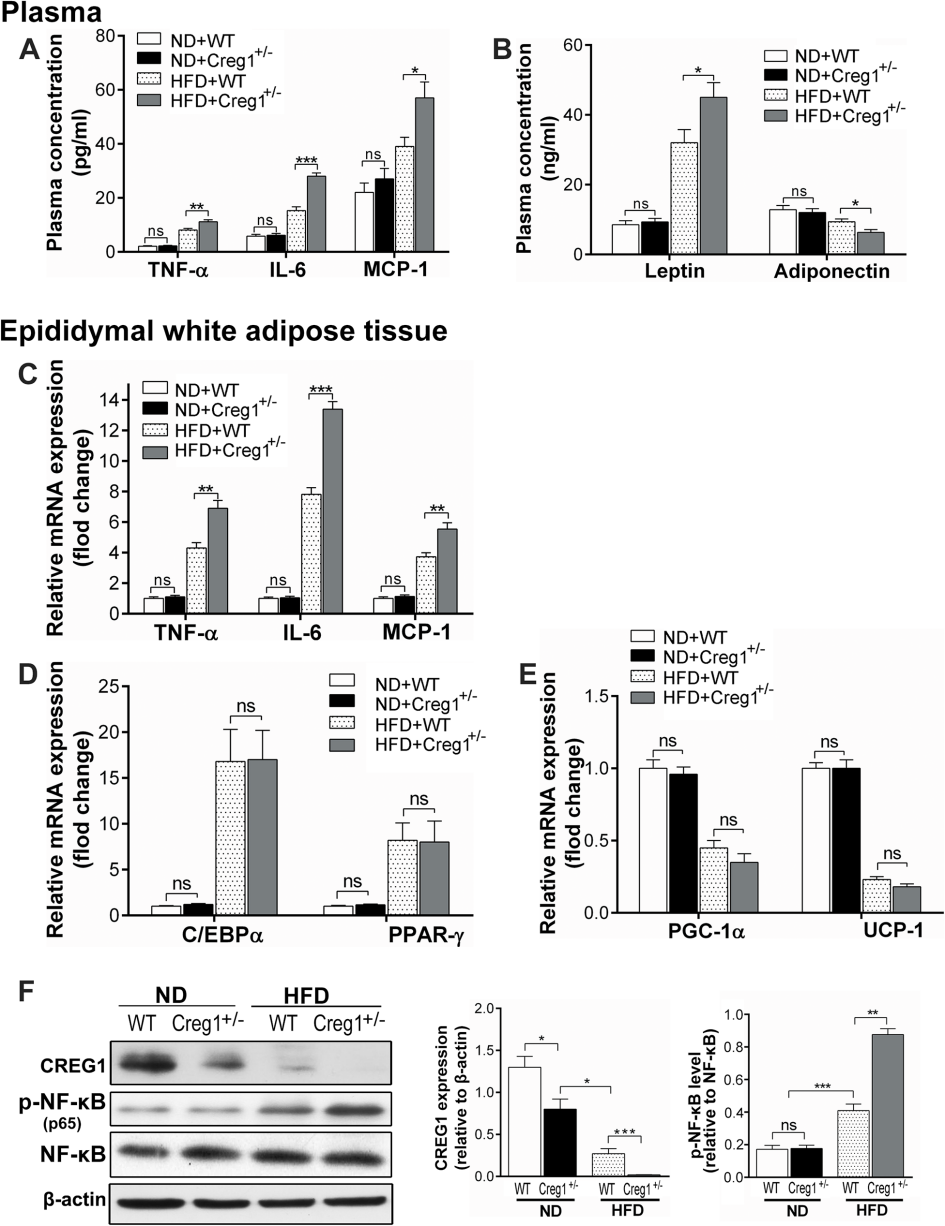
*Creg1*^*+/-*^ mice are sensitive to high fat diet (HFD)-induced systemic and adipose tissue inflammation. Plasma pro-inflammatory cytokines (**A**) and adipokines (**B**) were determined by ELISA method in wild type (WT) and *Creg1*^*+/-*^ mice on normal diet (ND) or HFD for 16 weeks (n = 5). In epididymal white adipose tissues (eWAT) from WT and *Creg1*^*+/*-^ mice fed on ND or HFD for 16 weeks, transcriptional expression of pro-inflammatory cytokines (**C**), adipogenesis related genes (**D**) and thermogenesis related genes (**E**) were assessed by Real-time PCR (n = 5). (**F**): CREG1 and phosphorylated NF-κB (p65) [p-NF-κB (p65)] were detected in tissue lysate of eWAT (left panel) by Western blot (left panel) (n = 3). Beta-actin and NF-κB were probed and used for normalization. The middle and right panel showed the quantification of CREG1 level relative to β-actin and p-NF-κB (p65) level relative to NF-κB respectively (n = 3 from three independent experiments). **Abbreviations**: TNF-α, tumor necrosis factor α; IL-6, interleukin 6; MCP-1, monocyte chemotactic protein 1; C/EBPα, CCAAT/enhancer-binding protein α; PPAR-γ, peroxisome proliferator-activated receptor γ; PGC-1α, peroxisome proliferator-activated receptor γ coactivator 1α; UCP-1, uncoupling protein 1.Values are presented as means ± SEM. ns, no significance. *P<0.05, **P<0.01, ***P<0.001.

Obesity associated chronic low-grade systemic inflammation is mainly mediated by WAT and plays an important role in the pathogenesis of insulin resistance and other metabolic abnormalities [27–29]. Thus we studied both systemic and adipose tissue inflammation. The plasma level of the pro-inflammatory cytokines (TNF-α and IL-6) (Fig 5A), the pro-inflammatory adipokine leptin and the anti-inflammatory adipokine adiponectin (Fig 5B) in *Creg1*^+/-^ mice was similar to that in the WT control on ND. The pro-inflammatory chemokine MCP-1 showed a tendency of increase in *Creg1*^+/-^ mice but failed to reach significance, suggesting that reduction of CREG1 may affect some cytokine expression even on a normal diet (Fig 5A). By contrast, on HFD, *Creg1*^+/-^ mice had significantly higher levels of TNF-α (*P*<0.01), IL-6 (*P*<0.001), MCP-1 (*P*<0.05), leptin (*P*<0.05) (Fig 5A) and a lower level of adiponectin (*P*<0.05) in plasma (Fig 5B). In epididymal WAT, we detected mRNA expression of the above pro-inflammatory cytokines by real-time PCR. The results were consistent with the findings in plasma. *Creg1* heterozygosity did not alter expression of TNF-α, IL-6 and MCP-1 fed with ND, but greatly increased their transcription on HFD as compared with WT counterparts (Fig 5C).

Adipogenesis and thermogenesis of WAT are also considered to be key components involved in pathogenesis of obesity [30–32]. Here we detected the expression of genes involved in adipogenesis (C/EBPα and PPAR-γ) [33] and thermogenesis (PGC-1α and UCP-1) [34]. Although HFD treatment significantly increased mRNA level of C/EBPα and PPAR-γ compared with ND, no significant difference was found between WT and *Creg1*^+/-^ mice (Fig 5D), suggesting that reduction of CREG1 has little if any effect on adipogenesis. As for thermogenesis, HFD treatment significantly decreased the mRNA levels of PGC-1α and UCP-1 in both genotypes. Although the mRNA levels of PGC-1α and UCP-1 in *Creg1*^+/-^ mice fed with HFD decreased more than the wild-type mice, the difference did not reach statistical significance (Fig 5E).

Inflammation of WAT is mediated mainly by the NF-κB and the mitogen-activated protein kinase (MAPK) pathway [35–37]. CREG1 has been reported to exert its anti-inflammatory effect through the NF-κB pathway [22, 23]. Therefore, we tested the hypothesis that the activation of NF-κB may mediate HFD-induced inflammation of WAT in *Creg1*^+/-^ mice. Western blot analysis of lysates of epididymal WAT showed that HFD significantly decreased CREG1 expression accompanied by increased the activation of NF-κB by phosphorylation [p-NF-κB (p65)] (*P*<0.001) in both WT and *Creg1*^+/-^ mice. Yet Creg1 haploinsufficiency caused a more robust, 2-fold increase of p-NF-κB (p65) (*P*<0.01) as compared with the WT control on HFD (Fig 5F). Altogether, these results indicate that *Creg1* haploinsufficiency increases HFD-induced systemic and adipose tissue inflammation without changing adipogenesis and thermogenesis of WAT.

### Depletion of CREG1 expression enhances inflammatory responses through the NF-κB pathway in 3T3-L1 adipocytes

**Fig 6:**
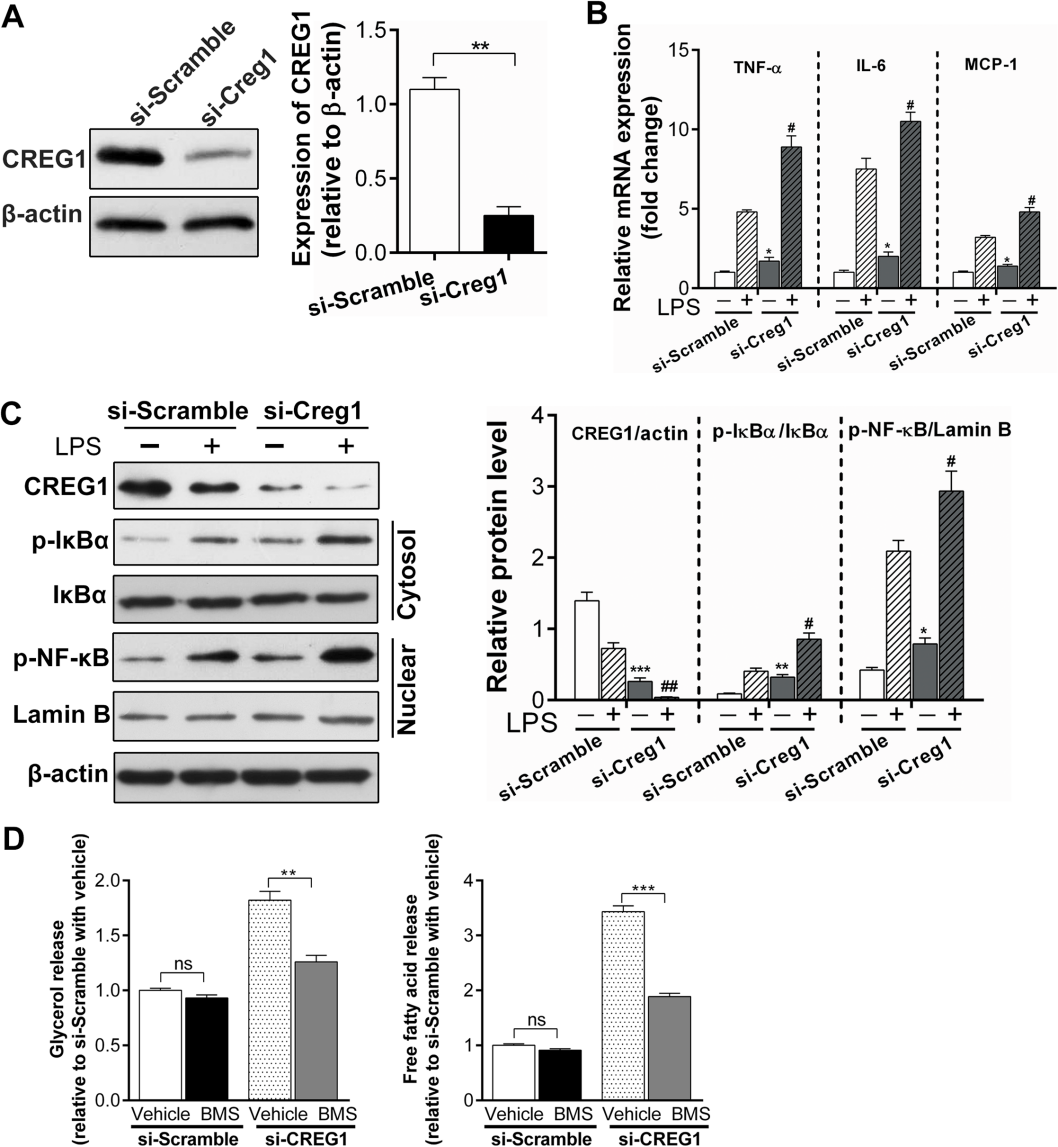
Depletion of CREG1 expression enhances inflammatory responses through the NF-κB pathway in 3T3-L1 adipocytes. (**A**): Knockdown of CREG1 by siRNA (siCreg1) in differentiated 3T3-L1 adipocytes were verified by Western blot (left panel) (n = 3). Beta-actin was used as loading control. The right panel showed the quantification of CREG1 relative to β-actin (n = 3 from three independent experiments). (**B**): Relative mRNA expression of TNF-α, IL-6 and MCP-1 in siCreg1 or si-Scramble treated 3T3-L1 cells ± 10 ng/ml LPS (n = 3). (**C**): Representative Western blots showed the effect of siCreg1 on pro-inflammatory signaling pathways (n = 3 replicated 3 times). The right panel showed the quantification of CREG1/actin, p-IκBα/IκBα and p-NF-κB/Lamin B. Values are presented as means ± SEM. ns, no significance. *P<0.05, **P<0.01 and ***P<0.001 compared with si-Scramble treated 3T3-L1 in the absence of LPS. #*P*<0.05 and ##*P*<0.01 compared with si-Creg1 treated 3T3-L1 in the presence of LPS. (**D**): Lipolysis were evaluated by measuring glycerol and free fatty acids released into the culture medium of siCreg1 or si-Scramble 3T3-L1 cells, which were treated with BMS-345541 (5 μmol/L), an NF-κB pathway inhibitor, or vehicle (DMSO) for 48 hours prior to lipolysis assessment. ns, no significance; **P<0.01 and ***P<0.001. **Abbreviations**: TNF-α, tumor necrosis factor-α; IL-6, interleukine-6; MCP-1, monocyte chemotactic protein-1; LPS, lipopolysaccharide; DMSO, dimethyl sulfoxide; BMS, BMS-345541.

To further investigate whether CREG1 is directly involved in the regulation of adipose inflammation, we employed the model of 3T3 L1 cell differentiation into adipocytes. We firstly silenced CREG1 expression in differentiated 3T3-L1 cells using siRNA method (si-Creg1). Western blotting showed that CREG1 was successfully knocked down by 90% at the protein level. Then si-Creg1 cells and the si-Scramble control cells were treated with or without LPS, and their expression of pro-inflammatory cytokines was detected. In the absence of LPS, the baseline level of the TNF-α, IL-6 and MCP-1 transcripts was higher in si-Creg1 cells than control cells (Fig 6B). Following LPS treatment, the expression of these pro-inflammatory cytokines was significantly up-regulated in both groups but to a much higher level in si-Creg1 cells (Fig 6B). Finally, we examined the effect of CREG1 knockdown on the activation of the NF-κB pathway. Silence of Creg1 in 3T3-L1 differentiated adipocytes activated the NF-κB pathway as evidenced by increased phosphorylation of IκBα and NF-κB in the cytosol and nuclear extract (Fig 6C). The activation of NF-κB by CREG1 insufficiency was observed both at the baseline level and after LPS challenge. These results suggest that CREG1 may regulate adipocyte inflammation through the NF-κB pathway. Interestingly, LPS treatment reduced CREG1 protein expression in both si-Creg1 and the control cells. The significance of this downregulation in LPS-induced inflammation warrants further investigation.

NF-κB pathway-triggered lipolysis plays essential role in HFD-induced obesity and insulin resistance[38]. To determine whether CREG1 is involved in this, lipolysis of differentiated 3T3 L1 cells transfected with si-Scramble or si-Creg1 was measured. Under basal conditions, CREG1 knockdown significantly stimulated lipolysis indicated by increased release of glycerol and free fatty acids into the culture medium (Fig 6D). In addition, the increased lipolysis in CREG1 knockdown cells could be blocked by the NF-κB pathway inhibitor BMS-345541. These results implicate that CREG1 negatively regulates lipolysis through the NF-κB pathway.

## Discussion

CREG1 has been shown to promote cell differentiation and survival [39, 40]. Previous studies mainly focused on its role in cardiovascular diseases and malignant tumors [21, 39, 41, 42]. Its implication in metabolic diseases has not been reported. In this study, we demonstrate for the first time that haplodeficiency of *Creg1* exacerbated HFD-induced obesity, insulin resistance and dyslipidemia. In addition, *Creg1*^+/-^ mice showed increased systemic and adipose tissue inflammation, possibly through activation of the NF-κB pathway.

In the present study, we used *Creg1*^+/-^ mice to explore the role of CREG1 because global knockout of *Creg1* leads to embryonic death around E7.5 (unpublished data), suggesting an essential role of *Creg1* in embryonic development. *Creg1*^+/-^ mice were viable and exhibited no apparently abnormal phenotype under normal feeding conditions, although CREG1 expression was reduced by ~50% in most tissues. Similar results were also found in many other heterozygote mice [43, 44], suggesting that haplodeficiency of *Creg1* is sufficient to maintain basal homeostasis. However, when fed with HFD, *Creg1*^+/-^ mice displayed significant body weight gain and visceral/subcutaneous fat adiposity compared with their WT littermates. Of note, the overt obese phenotype was not resulting from increased food intake, suggesting that *Creg1* haplodeficiency does not promote hyperphagia. Hyperphagia is regulated centrally by hypothalamus. Given that CREG1 is not expressed in the brain (the paralogous family member CREG2 is instead only expressed in the brain), it is unlikely that CREG1 has a direct impact on the hypothalamus. Another important factor regulating hyperphagia is leptin, which is secreted by WAT and acts through its receptor to provide a critical signal to hypothalamic neurocircuits to modulate food intake as well as energy expenditure[45]. We observed increased plasma leptin in *Creg1*^+/-^ mice. However, no hyperphagia was induced, suggesting leptin resistance in these mice.

High fat induced obesity is usually accompanied by insulin resistance and dyslipidemia[46, 47]. It’s not surprisingly to see that *Creg1*^+/-^ mice with such an obvious obese phenotype showed exacerbated insulin resistance indicated by increased fasting glucose and insulin level, higher HOMA-IR scores, lager AUC of GTT and ITT. The elevation of plasma TC, TG, LDL-C and FFA suggested dysfunction in lipid metabolism, possibly resulting from abnormalities of WAT and/or the liver. In fact, knockdown of CREG1 in 3T3-L1 cell-derived adipocytes leads to increased lipolysis, suggesting that CREG1 reduction-induced lipolysis may contribute to elevated plasma FFA.

Nutrient overload-induced obesity is associated with a state of low grade chronic systemic inflammation predominantly resulting from adipose tissue[48]. In this study, we observed that CREG1 haploinsufficiency in the heterozygous mice amplified HFD-induced systemic as well as WAT inflammation as indicated by increased expression of cytokines and chemokines. This is likely caused by the activation of the NF-κB signaling pathway because phosphorylation of the p65 subunit of NF-κB was increased in adipose tissues of *Creg1*^*+/-*^ mice on HFD. Furthermore, knockdown of CREG1 in differentiated adipocytes stimulated NF-κB phosphorylation and increased the expression of TNF-α, IL-6, and MCP-1. Importantly, depletion of CREG1 in adipocytes enhanced lipolysis and this effect could be blocked by NF-κB inhibition, suggesting that CREG1 suppresses lipolysis by inhibiting the NF-κB pathway. This inhibitory effect of CREG1 on lipolysis may raise the concern that it should produce a lean phenotype instead of obesity observed in *Creg1* haplodeficiency mice challenged with HFD. However, many studies have firmly demonstrated that basal lipolysis is increased in obese individuals[49, 50], which is consistent with our findings in vitro. Actually, the association between obesity and increased basal lipolysis is very complex and not fully understood. Increased insulin-induced lipolysis inhibition, enlarged fat cell size and elevated production and secretion of TNFα within adipose tissue may contribute to increased lipolysis in obese population[49, 50], which needs to be further studied.

Although we focused on white adipose tissue in the present study, we cannot rule out possible contributions of other tissues to the obese phenotype of *Creg1*^*+/-*^ mice on HFD. CREG1 is highly expressed in liver, heart and kidney which are very active in metabolism. Especially, the liver is a key organ participating in lipid metabolism. Therefore, development of tissue specific *Creg1* knockout mice will be necessary in the future study to clarify this issue.

## Supporting information

S1 Table Primer pairs. (PDF)

## References

[1] Zobel EH, Hansen TW, Rossing P, von Scholten BJ. Global Changes in Food Supply and the Obesity Epidemic. Current obesity reports. 2016;5(4):449–55. doi: 10.1007/s13679-016-0233-8 27696237 10.1007/s13679-016-0233-8

[2] Klil-Drori AJ, Azoulay L, Pollak MN. Cancer, obesity, diabetes, and antidiabetic drugs: is the fog clearing? Nature reviews Clinical oncology. 2016. 10.1038/nrclinonc.2016.120 27502359

[3] Mandviwala T, Khalid U, Deswal A. Obesity and Cardiovascular Disease: a Risk Factor or a Risk Marker? Current atherosclerosis reports. 2016;18(5):21 doi: 10.1007/s11883-016-0575-4 26973130 10.1007/s11883-016-0575-4

[4] Li L, Liu DW, Yan HY, Wang ZY, Zhao SH, Wang B. Obesity is an independent risk factor for non-alcoholic fatty liver disease: evidence from a meta-analysis of 21 cohort studies. Obesity reviews: an official journal of the International Association for the Study of Obesity. 2016;17(6):510–9. doi: 10.1111/obr.12407 10.1111/obr.12407 27020692

[5] Mitchell AB, Cole JW, McArdle PF, Cheng YC, Ryan KA, Sparks MJ, et al Obesity increases risk of ischemic stroke in young adults. Stroke. 2015;46(6):1690–2. doi: 10.1161/STROKEAHA.115.008940 25944320 10.1161/STROKEAHA.115.008940 PMC4458137

[6] Costa M, Garmendia ML, Corvalan C, Reyes M. The Presence and Duration of Overweight Are Associated with Low-Grade Inflammation in Prepubertal Chilean Children. Metabolic syndrome and related disorders. 2016;14(9):449–54. doi: 10.1089/met.2016.0055 27478998 10.1089/met.2016.0055

[7] Cooke AA, Connaughton RM, Lyons CL, McMorrow AM, Roche HM. Fatty acids and chronic low grade inflammation associated with obesity and the metabolic syndrome. European journal of pharmacology. 2016;785:207–14. doi: 10.1016/j.ejphar.2016.04.021 27083551 10.1016/j.ejphar.2016.04.021

[8] Chiang SH, Bazuine M, Lumeng CN, Geletka LM, Mowers J, White NM, et al The protein kinase IKKepsilon regulates energy balance in obese mice. Cell. 2009;138(5):961–75. doi: 10.1016/j.cell.2009.06.046 19737522 10.1016/j.cell.2009.06.046 PMC2756060

[9] Tam CS, Redman LM. Adipose tissue inflammation and metabolic dysfunction: a clinical perspective. Hormone molecular biology and clinical investigation. 2013;15(1):19–24. doi: 10.1515/hmbci-2013-0032 25436729 10.1515/hmbci-2013-0032 PMC6913892

[10] Veal E, Eisenstein M, Tseng ZH, Gill G. A cellular repressor of E1A-stimulated genes that inhibits activation by E2F. Molecular and cellular biology. 1998;18(9):5032–41. 9710587 10.1128/mcb.18.9.5032 PMC109088

[11] Kunita R, Otomo A, Ikeda JE. Identification and characterization of novel members of the CREG family, putative secreted glycoproteins expressed specifically in brain. Genomics. 2002;80(5):456–60. 12408961

[12] Schahs P, Weidinger P, Probst OC, Svoboda B, Stadlmann J, Beug H, et al Cellular repressor of E1A-stimulated genes is a bona fide lysosomal protein which undergoes proteolytic maturation during its biosynthesis. Experimental cell research. 2008;314(16):3036–47. doi: 10.1016/j.yexcr.2008.06.015 18621046 10.1016/j.yexcr.2008.06.015

[13] Di Bacco A, Gill G. The secreted glycoprotein CREG inhibits cell growth dependent on the mannose-6-phosphate/insulin-like growth factor II receptor. Oncogene. 2003;22(35):5436–45. doi: 10.1038/sj.onc.1206670 12934103 10.1038/sj.onc.1206670

[14] Sacher M, Di Bacco A, Lunin VV, Ye Z, Wagner J, Gill G, et al The crystal structure of CREG, a secreted glycoprotein involved in cellular growth and differentiation. Proceedings of the National Academy of Sciences of the United States of America. 2005;102(51):18326–31. doi: 10.1073/pnas.0505071102 16344469 10.1073/pnas.0505071102 PMC1317909

[15] Han Y, Deng J, Guo L, Yan C, Liang M, Kang J, et al CREG promotes a mature smooth muscle cell phenotype and reduces neointimal formation in balloon-injured rat carotid artery. Cardiovascular research. 2008;78(3):597–604. doi: 10.1093/cvr/cvn036 18267954 10.1093/cvr/cvn036

[16] Yan C, Fang P, Zhang H, Tao J, Tian X, Li Y, et al CREG1 promotes angiogenesis and neovascularization. Frontiers in bioscience (Landmark edition). 2014;19:1151–61. 24896341 10.2741/4272

[17] Tian X, Zhang N, Yan C, Nelsen J, Liu S, Kang J, et al CREG promotes vasculogenesis by activation of VEGF/PI3K/Akt pathway. Frontiers in bioscience (Landmark edition). 2014;19:1215–26. 24896346 10.2741/4277

[18] Liu J, Qi Y, Li S, Hsu SC, Saadat S, Hsu J, et al CREG1 Interacts with Sec8 to Promote Cardiomyogenic Differentiation and Cell-Cell Adhesion. Stem cells. 2016. 10.1002/stem.2434 27334848

[19] Sun M, Tian X, Liu Y, Zhu N, Li Y, Yang G, et al Cellular repressor of E1A-stimulated genes inhibits inflammation to decrease atherosclerosis in ApoE(-/-) mice. Journal of molecular and cellular cardiology. 2015;86:32–41. doi: 10.1016/j.yjmcc.2015.07.001 26163874 10.1016/j.yjmcc.2015.07.001

[20] Yan CH, Li Y, Tian XX, Zhu N, Song HX, Zhang J, et al CREG1 ameliorates myocardial fibrosis associated with autophagy activation and Rab7 expression. Biochimica et biophysica acta. 2015;1852(2):353–64. 25774384 10.1016/j.bbadis.2014.05.027

[21] Bian Z, Cai J, Shen DF, Chen L, Yan L, Tang Q, et al Cellular repressor of E1A-stimulated genes attenuates cardiac hypertrophy and fibrosis. Journal of cellular and molecular medicine. 2009;13(7):1302–13. doi: 10.1111/j.1582-4934.2008.00633.x 19413895 10.1111/j.1582-4934.2008.00633.x PMC4496144

[22] Peng CF, Han YL, Jie D, Yan CH, Jian K, Bo L, et al Overexpression of cellular repressor of E1A-stimulated genes inhibits TNF-alpha-induced apoptosis via NF-kappaB in mesenchymal stem cells. Biochemical and biophysical research communications. 2011;406(4):601–7. doi: 10.1016/j.bbrc.2011.02.100 21354106 10.1016/j.bbrc.2011.02.100

[23] Duan Y, Liu S, Tao J, You Y, Yang G, Yan C, et al Cellular repressor of E1A stimulated genes enhances endothelial monolayer integrity. Molecular biology reports. 2013;40(6):3891–900. doi: 10.1007/s11033-012-2373-6 23580165 10.1007/s11033-012-2373-6

[24] Song H, Yan C, Tian X, Zhu N, Li Y, Liu D, et al CREG protects from myocardial ischemia/reperfusion injury by regulating myocardial autophagy and apoptosis. Biochimica et biophysica acta. 2016. 10.1016/j.bbadis.2016.11.015 27840305

[25] Li Y, Yan CH, Han YL. CREG mediated adventitial fibroblast phenotype modulation: a possible therapeutic target for proliferative vascular disease. Medical hypotheses. 2012;79(1):95–7. doi: 10.1016/j.mehy.2012.04.011 22543074 10.1016/j.mehy.2012.04.011

[26] Muoio DM, Newgard CB. Obesity-related derangements in metabolic regulation. Annual review of biochemistry. 2006;75:367–401. doi: 10.1146/annurev.biochem.75.103004.142512 16756496 10.1146/annurev.biochem.75.103004.142512

[27] Lumeng CN, Saltiel AR. Inflammatory links between obesity and metabolic disease. The Journal of Clinical Investigation. 121(6):2111–7. doi: 10.1172/JCI57132 21633179 10.1172/JCI57132 PMC3104776

[28] Hotamisligil GS. Inflammation and metabolic disorders. Nature. 2006;444(7121):860–7. doi: 10.1038/nature05485 17167474 10.1038/nature05485

[29] Monteiro R, Azevedo I. Chronic Inflammation in Obesity and the Metabolic Syndrome. Mediators of Inflammation. 2010;2010:289645 doi: 10.1155/2010/289645 20706689 10.1155/2010/289645 PMC2913796

[30] Arce-Cerezo A, Garcia M, Rodriguez-Nuevo A, Crosa-Bonell M, Enguix N, Pero A, et al HMGA1 overexpression in adipose tissue impairs adipogenesis and prevents diet-induced obesity and insulin resistance. Scientific reports. 2015;5:14487 doi: 10.1038/srep14487 26411793 10.1038/srep14487 PMC4585969

[31] Bostrom P, Wu J, Jedrychowski MP, Korde A, Ye L, Lo JC, et al A PGC1-alpha-dependent myokine that drives brown-fat-like development of white fat and thermogenesis. Nature. 2012;481(7382):463–8. doi: 10.1038/nature10777 22237023 10.1038/nature10777 PMC3522098

[32] Peirce V, Carobbio S, Vidal-Puig A. The different shades of fat. Nature. 2014;510(7503):76–83. doi: 10.1038/nature13477 24899307 10.1038/nature13477

[33] Rosen ED, Spiegelman BM. Molecular Regulation of Adipogenesis. Annual Review of Cell and Developmental Biology. 2000;16(1):145–71. 10.1146/annurev.cellbio.16.1.145 11031233

[34] Inagaki T, Sakai J, Kajimura S. Transcriptional and epigenetic control of brown and beige adipose cell fate and function. Nat Rev Mol Cell Biol. 2016;advance online publication. 10.1038/nrm.2016.62 PMC4956538 27251423

[35] Yuan M, Konstantopoulos N, Lee J, Hansen L, Li ZW, Karin M, et al Reversal of obesity- and diet-induced insulin resistance with salicylates or targeted disruption of Ikkbeta. Science (New York, NY). 2001;293(5535):1673–7. 10.1126/science.1061620 11533494

[36] Sabio G, Das M, Mora A, Zhang Z, Jun JY, Ko HJ, et al A stress signaling pathway in adipose tissue regulates hepatic insulin resistance. Science (New York, NY). 2008;322(5907):1539–43. 10.1126/science.1160794 PMC2643026 19056984

[37] Lee BC, Lee J. Cellular and molecular players in adipose tissue inflammation in the development of obesity-induced insulin resistance. Biochimica et biophysica acta. 2014;1842(3):446–62. doi: 10.1016/j.bbadis.2013.05.017 23707515 10.1016/j.bbadis.2013.05.017 PMC3800253

[38] Baker RG, Hayden MS, Ghosh S. NF-κB, inflammation and metabolic disease. Cell metabolism. 2011;13(1):11–22. doi: 10.1016/j.cmet.2010.12.008 21195345 10.1016/j.cmet.2010.12.008 PMC3040418

[39] Veal E, Groisman R, Eisenstein M, Gill G. The secreted glycoprotein CREG enhances differentiation of NTERA-2 human embryonal carcinoma cells. Oncogene. 2000;19(17):2120–8. doi: 10.1038/sj.onc.1203529 10815803 10.1038/sj.onc.1203529

[40] Wang N, Han Y, Tao J, Huang M, You Y, Zhang H, et al Overexpression of CREG attenuates atherosclerotic endothelium apoptosis via VEGF/PI3K/AKT pathway. Atherosclerosis. 2011;218(2):543–51. doi: 10.1016/j.atherosclerosis.2011.08.002 21872252 10.1016/j.atherosclerosis.2011.08.002

[41] Piquemal D, Commes T, Manchon L, Lejeune M, Ferraz C, Pugnere D, et al Transcriptome analysis of monocytic leukemia cell differentiation. Genomics. 2002;80(3):361–71. 12213207 10.1006/geno.2002.6836

[42] Xu L, Liu J-M, Chen L-Y. CREG, a new regulator of ERK1/2 in cardiac hypertrophy. Journal of Hypertension. 2004;22(8):1579–87. 15257182 10.1097/01.hjh.0000133717.48334.cf

[43] Ali MI, Chen X, Didion SP. Heterozygous eNOS deficiency is associated with oxidative stress and endothelial dysfunction in diet-induced obesity. Physiological reports. 2015;3(12). 10.14814/phy2.12630 PMC4760452 26660551

[44] Li JJ, Ferry RJ Jr., Diao S, Xue B, Bahouth SW, Liao FF. Nedd4 haploinsufficient mice display moderate insulin resistance, enhanced lipolysis, and protection against high-fat diet-induced obesity. Endocrinology. 2015;156(4):1283–91. doi: 10.1210/en.2014-1909 25607895 10.1210/en.2014-1909 PMC4399314

[45] Park H-K, Ahima RS. Physiology of leptin: energy homeostasis, neuroendocrine function and metabolism. Metabolism: clinical and experimental. 2015;64(1):24–34. 25199978 10.1016/j.metabol.2014.08.004 PMC4267898

[46] Guo R, Zhang Y, Turdi S, Ren J. Adiponectin knockout accentuates high fat diet-induced obesity and cardiac dysfunction: role of autophagy. Biochimica et biophysica acta. 2013;1832(8):1136–48. doi: 10.1016/j.bbadis.2013.03.013 23524376 10.1016/j.bbadis.2013.03.013 PMC3796200

[47] Zhang Y, Yuan M, Bradley KM, Dong F, Anversa P, Ren J. Insulin-like growth factor 1 alleviates high-fat diet-induced myocardial contractile dysfunction: role of insulin signaling and mitochondrial function. Hypertension (Dallas, Tex: 1979). 2012;59(3):680–93. 10.1161/HYPERTENSIONAHA.111.181867 PMC3288378 22275536

[48] van der Heijden RA, Sheedfar F, Morrison MC, Hommelberg PPH, Kor D, Kloosterhuis NJ, et al High-fat diet induced obesity primes inflammation in adipose tissue prior to liver in C57BL/6j mice. Aging (Albany NY). 2015;7(4):256–67. 25979814 10.18632/aging.100738 PMC4429090

[49] Arner P. Control of lipolysis and its relevance to development of obesity in man. Diabetes/metabolism reviews. 1988;4(5):507–15. 3061758

[50] Arner P, Langin D. Lipolysis in lipid turnover, cancer cachexia, and obesity-induced insulin resistance. Trends in endocrinology and metabolism: TEM. 2014;25(5):255–62. doi: 10.1016/j.tem.2014.03.002 24731595 10.1016/j.tem.2014.03.002

