# Supplementary material for: CREG1 heterozygous mice are susceptible to high fat diet-induced obesity and insulin resistance
Source: PLoS One. 2017 May 1;12(5):e0176873. doi: 10.1371/journal.pone.0176873 (PMC5411056; doi:10.1371/journal.pone.0176873)
Supplement: S1 Table — (PDF) [file pone.0176873.s001.pdf]

S1 Table. Primer pairs

Gene name	Forward primer (5’-3’)	Reverse primer (5’-3’)	Size (bp)
Tumor necrosis factor α (TNF-α)	AAATGGGCTTTCCGAATTCA	CAGGGAAGAATCTGGAAAGGT	146
Interleukin 6 (IL-6)	GCTACCAAACTGGATATAATCAGGA	CCAGGTAGCTATGGTACTCCAGAA	78
Monocyte chemotactic protein-1 (MCP-1)	GCTGCTACTCATTCACCAGC	CTTCTTGGGGTCAGCACAGA	140
CCAAT/enhancer-binding protein (C/EBPα)	TCAGCTTACAACAGGCCAGG	ACACAAGGCTAATGGTCCCC	174
Peroxisome proliferator-activated receptor γ (PPAR-γ)	TTCGGAATCAGCTCTGTGGA	CCATTGGGTCAGCTCTTGTG	170
Peroxisome proliferator-activated receptor γ coactivator 1α (PGC-1α)	TAAATCTGCGGGATGATGGA	GTTTCGTTCGACCTGCGTAA	109
Uncoupling protein 1 (UCP-1)	GGATTGGCCTCTACGACTCA	TGCCACACCTCCAGTCATTA	107
Glyceraldehyde-3-phosphate dehydrogenase (GAPDH)	GTCGTGGAGTCTACTGGTGT	TGCTGACAATCTTGAGTGAG	183
